# Short disease-specific quality of life questionnaire for patients with uncomplicated symptomatic cholecystolithiasis: Gall-Questionnaire

**DOI:** 10.1093/bjsopen/zraf025

**Published:** 2025-04-09

**Authors:** Floris M Thunnissen, Daan J Comes, Verena N N Kornmann, Lindsey C F de Nes, Frans T W E van Workum, Sarah Z Wennmacker, Jose A E Custers, Philip R de Reuver

**Affiliations:** Department of Surgery, Radboud University Medical Centre, Nijmegen, The Netherlands; Department of Surgery, Radboud University Medical Centre, Nijmegen, The Netherlands; Department of Surgery, Maasziekenhuis Pantein, Boxmeer, The Netherlands; Department of Surgery, Maasziekenhuis Pantein, Boxmeer, The Netherlands; Department of Surgery, Canisius Wilhelmina Hospital, Nijmegen, The Netherlands; Department of Surgery, Radboud University Medical Centre, Nijmegen, The Netherlands; Department of Medical Psychology, Radboud University Medical Centre, Nijmegen, The Netherlands; Department of Surgery, Radboud University Medical Centre, Nijmegen, The Netherlands

Patient-reported outcome measures (PROMs) are often used to evaluate treatment success and measure the impact on quality of life (QoL). Several questionnaires, both generic and disease specific, are currently used to assess QoL in patients with abdominal pain and gallstones. However, these questionnaires are often lengthy or insufficiently specific to gallstone disease. A recent systematic review evaluated the available questionnaires and concluded that there is a need for a short, reliable instrument to assess disease-specific QoL in patients with abdominal symptoms and gallstones^1^. Therefore, a concise questionnaire was developed and validated, the Gall-Questionnaire (Gall-Q), to assess disease-specific QoL in patients with uncomplicated symptomatic cholecystolithiasis.

The study consisted of two phases: development and validation with evaluation of psychometric properties in a prospective multicentre cohort study. During phase 1, items for the Gall-Q were selected from four validated QoL questionnaires: the EuroQol-5 Dimension-3 Level questionnaire (EQ-5D-3L), the Gastrointestinal Quality of Life Index (GIQLI), the Gallstone Symptom List (GSL) and the Izbicki Pain Score (IPS). Items were discussed and selected during expert group meetings with surgeons, researchers and a behavioural scientist. Items were subdivided into three subscales: biliary symptoms, non-biliary abdominal symptoms and daily functioning. For validation of the Gall-Q, a validation study was conducted in two hospitals in The Netherlands. All consecutive patients with uncomplicated symptomatic gallstone disease were invited to complete questionnaires during their first outpatient clinic visit. The psychometric properties, including floor and ceiling effects (defined as 15% of participants achieving either the lowest or highest possible scores), internal consistency, structural validity and construct validity (that is convergent and divergent validity) were evaluated^2^. It was hypothesized that the questionnaire would correlate strongly (≥ 0.5) with the Patient Assessment of Upper Gastrointestinal Symptom Severity Index (PAGI-SYM) and moderately (0.3–0.5) with the Hospital Anxiety and Depression Scale (HADS).

The questionnaire consisted of 15 items (*Table 1*): five items on biliary symptoms, six items on non-biliary abdominal symptoms and four items on daily functioning. A total of 163 patients provided informed consent, and 156 patients completed all questionnaire items. Analyses of psychometric properties showed no floor or ceiling effects. In the 15-item questionnaire, two items (items 2 and 15) performed poorly regarding internal consistency and structural validity and were subsequently removed. The revised 13-item questionnaire demonstrated good internal consistency (Cronbach’s *α* 0.79). Exploratory factor analyses confirmed the three predefined subscales. As hypothesized, the questionnaire correlated strongly with the PAGI-SYM (0.737) and moderately with the HADS (0.509 on the anxiety scale and 0.337 on the depression scale), demonstrating good construct validity.

**Table 1:** The final items of the Gall-Questionnaire (Gall-Q)

1. How often have you experienced abdominal pain in the last 6 months?
2. When did the pain attacks start?*
3. How long does the pain usually last?
4. How often do you use pain medication to manage your pain?
5. How severe is your pain when it occurs? (Often measured using a numerical rating scale from 0 to 10.)
6. How often during the past 2 weeks have you had pain in the abdomen?
7. How often during the past 2 weeks have you had a feeling of fullness in the upper abdomen?
8. How often during the past 2 weeks have you had bloating (sensation of too much gas in the abdomen)?
9. How often during the past 2 weeks have you been troubled by constipation?
10. How often during the past 2 weeks have you been troubled by nausea?
11. How often during the past 2 weeks have you been troubled by heartburn?
12. How much does your disease-related pain interfere with your ability to work?
13. How often during the past 2 weeks have you been nervous or anxious about your illness?
14. Over the past week, have you woken up in the night?
15. During the past 2 weeks, how often have you been able to complete your normal daily activities (school, work, household)?*

*Item not included in final the 13-item questionnaire.

In addition to measuring surgical or disease-related complications, PROMs are crucial for accurately assessing symptoms and disease burden from the patient’s perspective. For symptomatic gallstone disease, this is especially relevant, as up to 40% of patients continue to experience pain for months or years after surgery^3^. Treatment effectiveness is often affected by coexisting disorders, such as functional dyspepsia or irritable bowel syndrome, which should be considered during initial consultations^4^. Unlike current questionnaires, the concise Gall-Q assesses both biliary and non-biliary abdominal complaints, making it suitable for routine clinical use.

This psychometric instrument is also a step forward in the shared decision-making process for patients with uncomplicated cholecystolithiasis. The uncertain outcome of treatment emphasizes the need for shared decision-making between patients and clinicians, especially in patients experiencing abdominal pain, gallstones and signs of functional gastrointestinal disorders^5^.

Accurately understanding patients’ symptoms through a targeted, disease-specific questionnaire is essential for advancing research and enhancing everyday patient care. A concise 13-item questionnaire with good reliability was developed to evaluate QoL in patients with symptomatic cholecystolithiasis.

## Data Availability

Data is available upon reasonable request.

## References

[1] Daliya P, Gemmill EH, Lobo DN, Parsons SL. A systematic review of patient reported outcome measures (PROMs) and quality of life reporting in patients undergoing laparoscopic cholecystectomy. Hepatobiliary Surg Nutr 2019;8:228–245 31245403 10.21037/hbsn.2019.03.16 PMC6561890

[2] Gagnier JJ, Lai J, Mokkink LB, Terwee CB. COSMIN reporting guideline for studies on measurement properties of patient-reported outcome measures. Qual Life Res 2021;30:2197–2218 33818733 10.1007/s11136-021-02822-4

[3] Comes DJ, Wennmacker SZ, Latenstein CSS, van der Bilt J, Buyne O, Donkervoort SC et al Restrictive strategy vs usual care for cholecystectomy in patients with abdominal pain and gallstones: 5-year follow-up of the SECURE Randomized Clinical Trial. JAMA Surg 2024;159:1235–1243 39167382 10.1001/jamasurg.2024.3080 PMC11339699

[4] de Jong JJ, Latenstein CSS, Boerma D, Hazebroek EJ, Hirsch D, Heikens JT et al Functional dyspepsia and irritable bowel syndrome are highly prevalent in patients with gallstones and are negatively associated with outcomes after cholecystectomy: a Prospective, Multicenter, Observational Study (PERFECT—Trial). Ann Surg 2022;275:e766–e772 32889877 10.1097/SLA.0000000000004453

[5] Alexander HC, Nguyen CH, Moore MR, Bartlett AS, Hannam JA, Poole GH et al Measurement of patient-reported outcomes after laparoscopic cholecystectomy: a systematic review. Surg Endosc 2019;33:2061–2071 30937619 10.1007/s00464-019-06745-7

